# Regulation of TFEB in human placental Cytotrophoblasts and Syncytiotrophoblasts

**DOI:** 10.14814/phy2.70383

**Published:** 2025-05-26

**Authors:** A. Mathew, J. S. Trausch‐Azar, C. Azar, M. Schuetz, M. R. Mahjoub, A. L. Schwartz

**Affiliations:** ^1^ Department of Pediatrics Washington University School of Medicine St. Louis Missouri USA; ^2^ Department of Medicine (Nephrology) Washington University School of Medicine St. Louis Missouri USA

**Keywords:** cytotrophoblast, degradation, placental, syncytiotrophoblast, ubiquitin

## Abstract

While cellular proteins exist in a dynamic state maintained by the balance of synthesis and degradation, there is a paucity of information on these processes in placental trophoblasts, including within cytotrophoblasts which differentiate into multi‐nucleate syncytiotrophoblasts. TFEB, a transcription factor with a myriad of cellular activities, is one of the most abundant genes expressed in syncytiotrophoblasts compared to cytotrophoblasts. TFEB is localized to the nucleus of human BeWo differentiated syncytiotrophoblasts and to the cytoplasm of the undifferentiated cytotrophoblasts. Within both the cytotrophoblasts and syncytiotrophoblasts, TFEB exists in subcellular compartments as both phosphorylated and unphosphorylated forms and translocates between cytoplasm and nucleus upon amino acid starvation/refeeding. Endogenous TFEB and endogenous phospho‐TFEB are both rapidly (*t*_1/2_ ~ 2–3 h) degraded via the ubiquitin proteasome system in cytotrophoblasts and in syncytiotrophoblasts. These results suggest dynamic regulatory processes during trophoblast development/differentiation.

## 1 INTRODUCTION

Cellular proteins exist in a dynamic state maintained by the balance of synthesis and degradation. The ubiquitin proteolytic pathway is responsible for the degradation of the bulk of cellular proteins, including regulatory, misfolded/denatured, and short‐lived proteins, including transcription factors and oncoproteins (Ciechanover et al., 1991; Schwartz & Ciechanover, 2009). Ubiquitin‐mediated proteolysis involves the covalent attachment of multiple ubiquitin molecules to the protein substrate. Degradation of the targeted protein is carried out by the 26S proteasome. The system functions both in the cytoplasm as well as within the nucleus (Enam et al., 2018; Floyd et al., 2001; Franić et al., 2021; Lingbeck et al., 2003).

During development, the mammalian placenta, which is essential for fetal development and survival, undergoes remarkable differentiation in which single cytotrophoblast cells fuse to form the mature circum‐villous lining of syncytiotrophoblasts. The molecular basis of this complex developmental program is incompletely understood, but involves several essential proteins including syncytin‐1, syncytin‐2, and their receptors SLC1A5 and MFSD2A (Huppertz & Gauster, 2011).

As several short‐lived proteins are present in placental trophoblasts, we examined the degradation of transcription factor EB (TFEB) in BeWo cytotrophoblasts and their differentiated syncytiotrophoblasts. TFEB is a basic‐helix–loop–helix, leucine zipper (bHLH‐Zip) protein involved in organelle biogenesis and cellular metabolic control. TFEB is abundantly expressed in the placenta, especially in syncytiotrophoblasts (Uhlén et al., 2015); see The Human Protein Atlas (Nakashima et al., 2020). TFEB‐null mice die at ~e10 with disrupted vascularization of the placenta (Steingrímsson et al., 1998). Cesana et al. (2024) have recently shown that TFEB is involved in functional syncytiotrophoblast formation. In addition, tissue‐specific TFEB deletions have provided insights into metabolism in both liver and skeletal muscle (Napolitano & Ballabio, 2016).

TFEB, as a master regulator of lysosomal function, is essential to normal lysosomal processes including endocytosis and autophagy (Napolitano & Ballabio, 2016). TFEB also plays a pivotal role in organelle biogenesis, cell metabolism, lysosomal biogenesis, lipid metabolism, and energy metabolism (Settembre et al., 2013). Sardiello et al. (2009) showed that TFEB overexpression results in increases in lysosome number, concentration of lysosomal enzymes, and lysosomal catabolic function. Similarly, Palmieri et al. (2011) showed that TFEB binds the promoter regions of multiple autophagy genes, resulting in induction of autophagosome biogenesis and fusion with lysosomes, as well as enhanced degradation of autophagy substrates including long‐lived proteins.

Regulation of TFEB activity and cellular levels is controlled via phosphorylation, subcellular localization, and degradation (Napolitano et al., 2018; Puertollano et al., 2018; Tan et al., 2022). Regulatory phosphorylation occurs at numerous serine residues mediated in large part via mTOR. Phosphorylated TFEB is largely cytoplasmic, while TFEB within the nucleus is non‐phosphorylated (Puertollano et al., 2018; Tan et al., 2022). Recently, Sha et al. (2017) demonstrated that, in HeLa, HEK, and MEF cells, TFEB degradation is via the ubiquitin‐proteasome pathway and that inactive, phosphorylated cytoplasmic TFEB was preferentially targeted for degradation via the ubiquitin E3 ligase, STUB1.

Herein, we investigated the ubiquitin proteasome‐mediated degradation of TFEB in BeWo human cytotrophoblasts and syncytiotrophoblasts. We show that endogenous full‐length phosphorylated TFEB is localized to the cytoplasm of cytotrophoblasts and unphosphorylated TFEB is localized largely to the nucleus of syncytiotrophoblasts. Regardless of location and phosphorylation state, TFEB responds to amino acid starvation and refeeding. Endogenous TFEB and phospho‐TFEB in cytotrophoblasts and in syncytiotrophoblasts are rapidly degraded in a ubiquitin proteasome‐dependent manner. These findings provide a basis for further elucidation of trophoblast TFEB biology during development.

## 2 METHODS

### 2.1 Cell culture and chemical reagents

The HeLa cell line was obtained from the American Type Culture Collection. The BeWo b30 cell line, developed in the Schwartz lab, has been previously described (Azar et al., 2018, 2021; Wice et al., 1990). HeLa cells were propagated as described earlier (Trausch‐Azar et al., 2004). Briefly, cells were cultured in growth medium, Dulbecco's modified Eagle's medium (Gibco 11,965,084, (4.5 g/L D‐glucose)) supplemented with 10% fetal bovine serum and maintained in a humidified chamber at 37°C with 5% CO_2_. BeWo cells were propagated as described earlier (Azar et al., 2018). Briefly, cells were cultured in F‐12 K Nutrient Mix, Kaighn's Modification (Gibco 21,127,022, D‐glucose 1260 mg/L), 10% fetal bovine serum and 2 mM glutamine (Wice et al., 1990). Differentiation of BeWo cytotrophoblasts to syncytiotrophoblasts was performed by 72 h incubation with 100 μM forskolin (Sigma F3917) as described earlier (Azar et al., 2018, 2021).

Antibody reagents were as follows. Rabbit anti‐human TFEB was from Bethyl (#A303‐673A) and rabbit anti‐human TFEB‐phosphoSer122 and anti‐human TFEB‐phosphoSer211 were from Cell Signaling (#86843/#87932S and #36781, respectively). Rabbit anti‐human antibodies were actin (Sigma #A5441), alpha‐tubulin (Sigma #T6199), lamin B1 (Abcam #ab133741), as well as hCGβ (Abcam ab9376), and rat monoclonal anti‐human E cadherin (Santa Cruz #sc59778). Secondary antibodies included goat anti‐rabbit‐HRP (Cell Signaling Technologies #7074S), goat anti‐rat monoclonal‐HRP (Invitrogen #A11077), goat anti‐rat‐AlexaFluor568 (Invitrogen #A11077), and goat anti‐rabbit‐AlexaFluor488 (Invitrogen #A11008).

MG132 (20 μM; Peptides International) was prepared as a 10 mM stock solution in DMSO. Torin (250 nM; Calbiochem) was prepared as a 250 μM stock solution in DMSO.

### 2.2 Subcellular fractionation

HeLa cells or BeWo cells ± forskolin treatment were either treated with ± 250 nM Torin for 1 h or were starved/refed and then analyzed by cell fractionation. Starvation/refeeding was as follows: cells were refed with fresh growth media for 2 h, thereafter cells were incubated in starvation media (EBSS, Gibco #24010043) for 1 h, and those cells which were refed were incubated for 2 h in fresh growth media. At the appropriate times, cells were harvested and processed as per Roczniak‐Ferguson et al. (2012). Briefly, cells were rinsed in PBSc, harvested in ice‐cold hypotonic buffer homogenized with 20 strokes of Dounce homogenizer (5 loose; 15 tight), yielding “whole cell extract”. The remainder was centrifuged at 4°C for 5 min at 13,200 rpm. The supernatant (“post nuclear supernatant”) was removed. The high salt buffer‐resuspended nuclear pellet (“nuclear extract”) was then sonicated. Aliquots of each fraction were placed in Laemmli sample buffer and equal cell‐equivalents were analyzed via Western blot together with controls (alpha‐tubulin and lamin B1).

### 2.3 Immunofluorescence

Subcellular localization of TFEB was determined by indirect immunofluorescence in HeLa and BeWo cells as described previously (Azar et al., 2018, 2021; Trausch‐Azar et al., 2015). Briefly, cells were seeded onto glass cover slips in six‐well dishes and treated with forskolin as described. At harvest, cells were washed with PBSc, a phosphate‐buffered saline solution (PBSa; Corning) supplemented with 100 mM CaCl_2_ and 50 mM MgCl_2_, fixed in 4% paraformaldehyde (Electron Microscopy Sciences, Hatfield, PA), quenched in 0.1 M ethanolamine (pH 8.0), and permeabilized in 1% Triton X‐100 (Sigma). After blocking in 1% BSA/PBSc/0.1% Tween 80, subcellular localization was then determined using rabbit polyclonal anti‐TFEB (1:1000 dilution) and rat anti‐human E‐cadherin (1:500) followed by incubation with AlexaFluor 488 goat anti‐rabbit IgG and AlexaFluor 568 goat anti‐rat (Invitrogen), thereafter incubated with DAPI (Sigma D9542) and mounted using Mowiol (Sigma 475,904) containing 2.5% 1,4‐diazo‐bicyclo‐[2.2.2]‐octane (DABCO, Sigma D2522). Cells were observed using a Zeiss Axioscope microscope, and images were taken using a Zeiss AxioCam digital camera.

### 2.4 Confocal microscopy

Images of fixed cells were captured using a Nikon Eclipse Ti‐E inverted confocal microscope equipped with a 20X air objective lens (Nikon, Melville, NY). A series of digital optical fields were captured using an Andor Neo‐Zyla CMOS camera at room temperature. Optical fields were stitched together and reconstructed using Nikon Elements AR 4 Software, as described previously (Azar et al., 2021).

### 2.5 Quantification of TFEB staining of trophoblasts

Confocal microscopic images of triple immuno‐labeled cells were quantified by scoring each cell as a single cell or within a syncytium of at least 2 nuclei without E‐cadherin junction staining between them, as described previously (Azar et al., 2018, 2021). Cells were scored as TFEB stained predominantly within the cytoplasm (C), predominantly within the nucleus (N) or present in both cytoplasm and nucleus (C/N). Over 5300 cells from three independent experiments were scored and data are presented as mean ± SEM.

### 2.6 Protein analysis

For cellular protein analysis, BeWo or HeLa cells were rinsed in PBSc at 4°C and thereafter incubated for 20 min at 4°C with fresh lysis buffer PBSc containing 5% Igepal (Sigma CA‐630), 1 mM EDTA, 1 mM DTT, and Complete Protease Cocktail (Roche 11,834,170,001) followed by centrifugation (15 min at 13,000 × *g* at 4°C) and the resultant supernatant was stored at −20°C, as described previously (Trausch‐Azar et al., 2015).

### 2.7 Determination of protein half‐life

As described previously (Lingbeck et al., 2003; Trausch‐Azar et al., 2010), HeLa or BeWo cells (± forskolin treatment for 72 h) were incubated with cycloheximide (CHX) (100 μg/mL; Sigma C1988) to inhibit further protein synthesis. MG132 (20 μM; Peptides International 3175‐V) was added along with CHX as noted. The cells were lysed after 0, 2, 4, and 6 h (for CHX alone) or 0, 4, and 6 h (for CHX plus MG132) in lysis buffer (see above) for at least 30 min, after which cells were centrifuged at 13,000 rpm for 10 min at 4°C in an Eppendorf microcentrifuge to remove cellular debris. The lysates were mixed with Laemmli sample buffer (Bio‐Rad), and equal amounts of sample were run on a 10%Tris–HCl gel (Bio‐Rad) followed by electroblotting onto nitrocellulose (Amersham). For the detection of TFEB, the blots were probed with polyclonal anti‐TFEB (1:1000 dilution) followed by incubation with a secondary horseradish peroxidase‐conjugated antibody and detection by chemiluminescence (Pierce ECL‐Western Blotting Substrate Thermo Fisher 32,106). For the detection of phospho‐TFEB, membranes were stripped using ReBlot Plus Strong Antibody Stripping Solution (Millipore). Blots were then probed with anti‐phospho‐TFEB, incubated with a secondary horseradish peroxidase‐conjugated antibody, and detected by chemiluminescence (SuperSignal West Femto—Thermo Fisher 34,095). The resulting bands were quantitated using ImageJ, and the data were graphed using the Excel graphing program (Microsoft). The protein degradation rate is expressed as half‐life (*t*_½_), the time for degradation of 50% of the protein. Half‐life data reported were evaluated in 4 independent determinations.

### 2.8 Statistical analysis

Statistical analysis, SD, SEM, and *t*‐test comparisons were performed using Prism software. Significance is *p* < 0.05.

## 3 RESULTS

**FIGURE 1:**
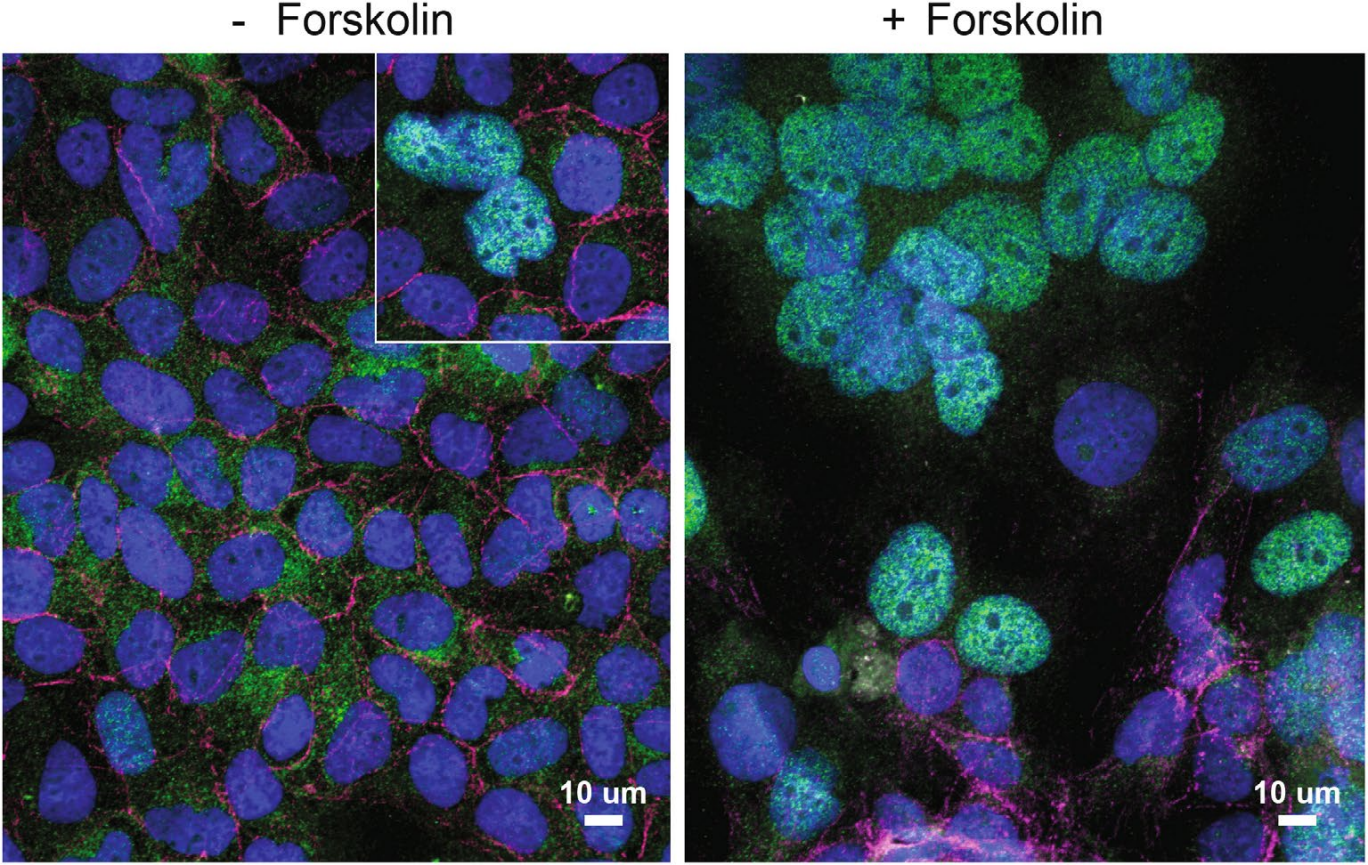
Triple‐label Immunofluorescence Localization of BeWo Cytotrophoblasts (left) and Syncytiotrophoblasts (right). BeWo cells were treated with vehicle control (left) or with forskolin (right) and after 72 h cells were fixed and immunostained. Nuclei were stained with DAPI (blue), cell junctions were stained with anti‐E cadherin (red) and TFEB was stained with anti‐TFEB (green). Inset in left panel is of naturally occurring syncytia. Bars in lower right represent 10 μm.

**FIGURE 2:**
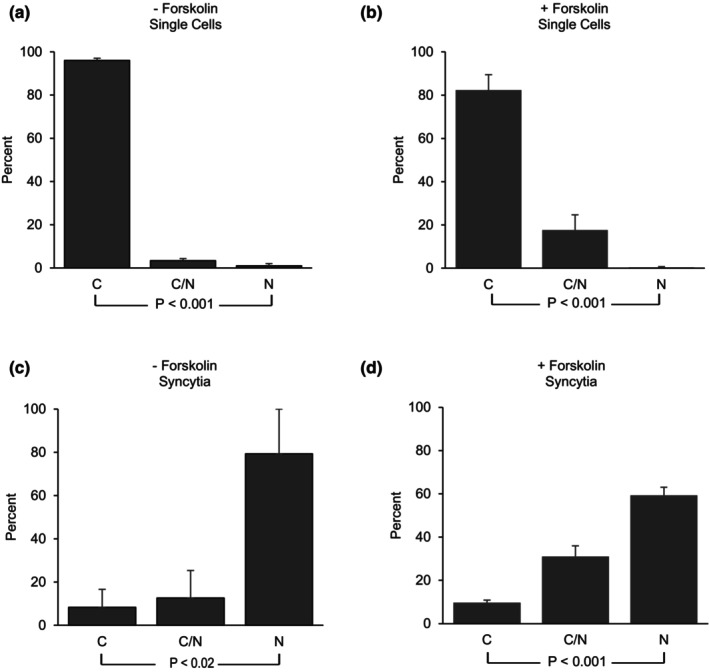
Quantification of TFEB Localization in BeWo Cytotrophoblasts and Syncytotrophoblasts. Triple immunostained cells as seen in Figure 1 were scored for TFEB expression as predominantly cytoplasmic (C), predominantly nuclear (N) or present in both cytoplasm and nucleus (C/N) in single cells (a, b) or within syncytia (c, d) in cells treated with vehicle alone (a, c) or with forskolin (b, d). In cells treated with vehicle alone, greater than 99% of cells were single cells, whereas in cells treated with forskolin 58% of nuclei were in syncytia and 42% were in single cells. 5329 cells were scored from 3 independent experiments (panel a: 3785; b: 629; c: 49; d: 866). Data are displayed as mean ± SEM of the 3 experiments. Statistical comparison of C versus N: A, b, d: *p* < 0.001; c: *p* < 0.02.

In normal term human placenta, TFEB is expressed predominately in the syncytiotrophoblast lining the microvilli (Human Protein Atlas, Nakashima et al., 2020; Thul et al., 2017; Uhlén et al., 2015). Using RNASeq, Azar et al., (2018) showed that TFEB was the 38th most up‐regulated gene expressed in human placental syncytiotrophoblasts compared to cytotrophoblasts. Human BeWo cells are a well‐defined model of cytotrophoblast to syncytiotrophoblast differentiation in vitro (Azar et al., 2018, 2021; Wice et al., 1990) and express abundant TFEB (see Human Protein Atlas). As seen in Figure 1, TFEB is localized predominately in the cytoplasm of BeWo cytotrophoblasts and in the nucleus of BeWo syncytiotrophoblasts. In BeWo cells growing in culture (Figures 1 and 2a,c) greater than 99% of cells are single cytotrophoblasts. Rare (i.e. less than 1%) multi‐nucleated syncytia are present (Figures 1 and 2c). Single cytotrophoblast cells demonstrate TFEB predominately within the cytoplasm. In contrast, the rare multinucleate naturally occurring syncytia demonstrates TFEB predominately within the nucleus (Figures 1 and 2a). The treatment of BeWo cells with forskolin results in marked fusion of cytotrophoblasts to form multinucleated syncytia (i.e., syncytiotrophoblasts) as originally reported by Wice et al., (1990). hCGβ is abundantly expressed in syncytiotrophoblasts and in BeWo cells treated with forskolin (see Wice et al., 1990; Azar et al., 2018; and Figure S1). As seen in Figures 1 and 2b,d, following 72 h incubation with 100 μM forskolin, ~60% of cell nuclei are within syncytia, similar to earlier reports of 40%–60% of cell nuclei within syncytia upon forskolin‐treatment (Azar et al., 2021; Orendi et al., 2010). In BeWo cells treated with forskolin, TFEB was predominately localized to the cytoplasm in the single cytotrophoblasts (Figures 1 and 2b). In contrast, TFEB was predominately within the nuclei of syncytiotrophoblasts (Figures 1 and 2d). Thus, TFEB localization is largely cytoplasmic in cytotrophoblasts and nuclear in syncytiotrophoblasts.

**FIGURE 3:**
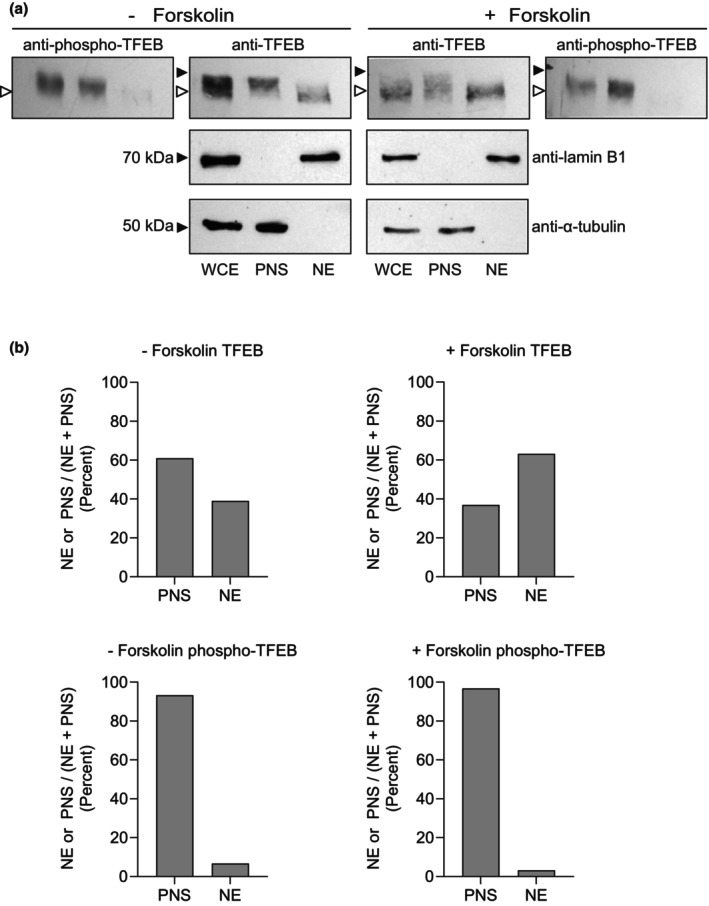
TFEB Localization in Cell Fractions of BeWo Cytotrophoblasts and Syncytiotrophoblasts. BeWo cells were treated with vehicle alone or forskolin for 72 h and then harvested following which whole cell extract (WCE), post‐nuclear supernatant extract (PNS, cytoplasm) and nuclear extract (NE) were prepared and analyzed via Western blot with anti‐TFEB antibody. Blots were then reprobed with anti‐phospho TFEB antibodies as indicated. Controls of anti‐lamin B1 and anti‐alpha tubulin are shown. The 75 kDa marker is indicated by closed triangle. The open triangle denotes lower molecular weight (unphosphorylated) TFEB.

Cellular fractionation studies were used to independently examine TFEB localization in BeWo cells treated without and with forskolin (i.e., cytotrophoblasts and syncytiotrophoblasts). Fractionation of HeLa cells using the procedure of Roczniak‐Ferguson et al. (2012) results in nearly complete separation of cytoplasmic markers (e.g., alpha‐tubulin) in the post‐nuclear supernatant fraction and nuclear markers (e.g., lamin B1) in the nuclear fraction (Figure S2). TFEB is largely in the post‐nuclear supernatant (i.e., cytoplasm) of growing HeLa cells (Figure S2) as shown earlier by Vega‐Rubin‐de‐Celis et al. (2017). The TFEB band extends from ~65 to ~75 kDa and represents both non‐phosphorylated TFEB (lower apparent molecular weight bands) and phosphorylated TFEB (higher apparent molecular weight bands) (Martina et al., 2012). Results in BeWo cells in the absence of forskolin (i.e., cytotrophoblasts) (Figure 3) demonstrate TFEB predominately in the post nuclear supernatant (i.e. cytoplasm) while in the presence of forskolin (i.e. ~60% syncytiotrophoblasts/~40% cytotrophoblasts), TFEB is both in the post‐nuclear supernatant and nuclear fractions (Figure 3). In both cytotrophoblasts and syncytiotrophoblasts, the TFEB in the post‐nuclear supernatant (cytoplasm) is of greater apparent molecular weight than the TFEB within the nuclear fractions. Reprobing these blots with anti‐phospho‐TFEB revealed phospho‐TFEB in the BeWo cells (± forskolin) in the post‐nuclear supernatant and virtually absent from the nuclear fractions (Figure 3). These observations are consistent with those of others in HeLa cells (Martina et al., 2012; Vega‐Rubin‐de‐Celis et al., 2017).

**FIGURE 4:**
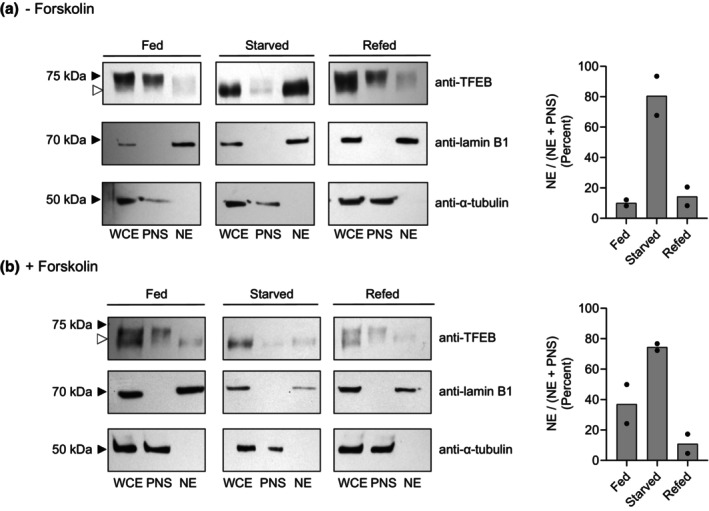
TFEB Localization Following Amino Acid Starvation and Refeeding in BeWo Cytotrophoblasts and Synctiotrophoblasts. Following treatment ± forskolin, cells were fed with complete media for 2 h, amino acid starved in EBSS for 1 h, and then refed for 1 h with complete media, as indicated. Thereafter, cells were harvested, fractionated, run on gels, and blots thereafter probed with anti‐TFEB as in Figure 3. Controls and markers are as in Figure 3. Data from individual experiments are displayed as circles and the mean as a bar.

We next examined the effect of amino acid starvation on the cellular localization of TFEB. As has been previously shown in HeLa cells, 1 h amino acid starvation results in TFEB dephosphorylation and translocation from the cytoplasm to the nucleus and refeeding with amino acids results in TFEB rephosphorylation and translocation back into the cytoplasm. Similar results were seen in BeWo cells. As seen in Figure 4, in the absence of forskolin (i.e., in cytotrophoblasts) TFEB is largely in cytoplasm and of higher molecular weight. Upon amino acid starvation, TFEB is now largely nuclear and of lower molecular weight. Refeeding cells with amino acids results in TFEB translocation back to the cytoplasm and of higher molecular weight (Figure 4). BeWo cells in the presence of forskolin (~40% single cells, ~60% multi‐nucleated syncytiotrophoblasts) demonstrated TFEB in both the post nuclear supernatant (i.e., cytoplasm) as well as in the nucleus. In the nuclear fraction the TFEB is of lower apparent molecular weight (Figure 4), similar to that seen in Figure 3. In addition, the fraction of “nuclear plus post nuclear supernatant” which is “nuclear” is greater in BeWo cells in the presence of forskolin (i.e., in syncytiothrophoblasts) than in its absence (i.e., in cytotrophoblasts) (Figure 4). Thus, similar to earlier studies in HeLa cells, in BeWo cytotrophoblasts and syncytiotrophoblasts, TFEB appears to translocate between cytoplasm and nucleus with the cytoplasmic TFEB largely of higher apparent mwt (i.e., phosphorylated) and nuclear TFEB of lower apparent mwt (i.e., unphosphorylated).

Further, we explored the fraction of cellular TFEB which is phospho‐TFEB. The phospho‐TFEB fraction in BeWo cells (± forskolin) was examined in the fed versus starved state (Figure S4). In BeWo cells (−forskolin) phospho‐TFEB is 32% of total TFEB, and in BeWo cells (+forskolin) phospho‐TFEB is 4% of total TFEB. In BeWo cells (−forskolin) starvation reduces phospho‐TFEB to 10% of total TFEB, and in BeWo cells (+forskolin) starvation reduces phospho‐TFEB to 1% of total TFEB.

**FIGURE 5:**
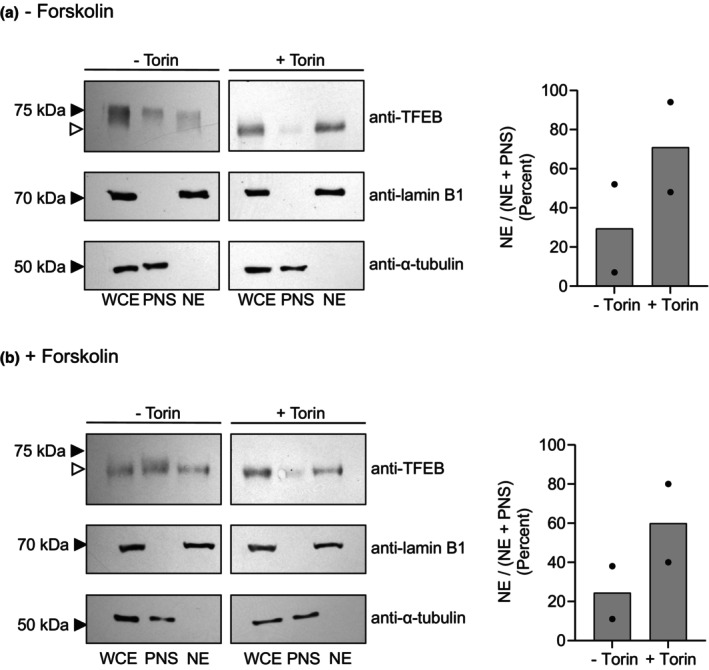
TFEB Localization Following Torin Treatment in BeWo Cytotrophoblasts and Synctiotrophoblasts. Following treatment ± forskolin, cells were incubated without or with 250 nM Torin, as indicated. Thereafter cells were harvested, fractionated, run on gels, and blots thereafter probed with anti‐TFEB as in Figure 3. Controls and markers are as in Figure 3. Data from individual experiments are displayed as circles and the mean as a bar.

These results are supported by studies on the effect of Torin, an mTOR inhibitor, on TFEB localization. As has been seen previously in HeLa cells, 1 h incubation with 250 nM Torin results in TFEB translocation from the cytoplasm to the nucleus (Figure S3). Similar results were seen in BeWo cytotrophoblasts and syncytiotrophoblasts (Figure 5) where Torin treatment results in TFEB translocation to the nucleus and of lower apparent molecular weight. Taken together, these results with cell fractionation, starvation/refeeding, and Torin treatment support the cytoplasmic–nuclear shuttling of TFEB in BeWo cells.

**FIGURE 6:**
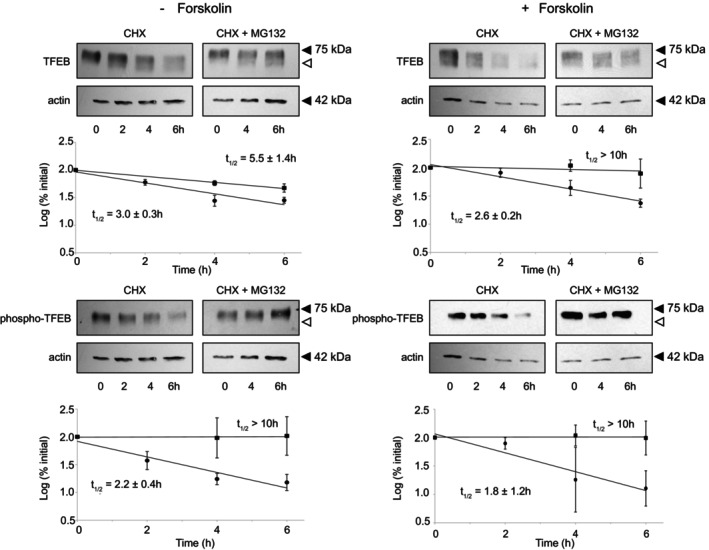
Degradation of Endogenous TFEB and Phospho‐TFEB in BeWo Cytotrophoblasts and Syncytiotrophoblasts. Following 72 h treatment with vehicle alone (cytotrophoblasts) or 100 μM forskolin (syncytiotrophoblasts), cells were incubated with 100 μg/mL cycloheximide (CHX) in the absence or presence of 20 μM MG132. At 0, 2, 4 and 6 h (CHX) and 0, 4 and 6 h (CHX + MG132) cells were harvested and analyzed via Western blot with anti‐TFEB antibody and subsequently reprobed with anti‐phospho‐TFEB as described in Methods. The TFEB and phospho‐TFEB were quantified as described in Methods and displayed in the Figure, CHX alone (●), CHX + MG132 (■). Half‐lives were calculated as indicated (mean ± SEM of 4 independent experiments). Upper panels are representative Western blots with anti‐TFEB, anti‐phospho‐TFEB and anti‐Actin, as control. Statistical comparison of TFEB and of phospho‐TFEB *t*_1/2_ (−forskolin versus + forskolin) are not significant (*p* > 0.05). Statistical comparison of TFEB and of phospho‐TFEB (− forskolin versus + forskolin) at 2, 4 and 6 h are each not significant (*p* > 0.05).

To examine the role of the ubiquitin‐proteasome pathway in the degradation of TFEB, we first examined the degradation of endogenous TFEB in HeLa cells. We incubated cells with cycloheximide for various times in the absence or presence of MG132, a potent proteasomal inhibitor, as described previously, and followed the fate of TFEB over 6 h (Trausch‐Azar et al., 2010, 2015). As seen in Figure S5, the degradation of endogenous TFEB in HeLa cells is rapid with *t*_1/2_ ~ 3 h and is inhibited by the presence of MG132. Similarly, in BeWo cells, the degradation of endogenous TFEB in cytotrophoblasts (i.e., in the absence of forskolin) is rapid (t_1/2_ ~ 3 h) and is inhibited by MG132. In BeWo syncytotrophoblasts (i.e., in the presence of forskolin) TFEB degradation is also rapid (*t*_1/2_ ~ 2.6 h) and is inhibited by MG132 (Figure 6). We next examined the degradation of phospho‐TFEB. In BeWo cells, the degradation of endogenous phospho‐TFEB in cytotrophoblasts is rapid (*t*_1/2_ ~ 2.2 h) and is inhibited by MG132. In BeWo syncytiotrophoblasts, phospho‐TFEB degradation is also rapid (*t*_1/2_ ~ 1.8 h) and is inhibited by MG132 (Figure 6). The degradation of phospho‐TFEB appears to be more rapid than that of TFEB in both cytotrophoblasts and syncytiotrophoblasts. These data thus support the ubiquitin proteasome‐dependent degradation of endogenous TFEB in both BeWo cytotrophoblasts and syncytiotrophoblasts.

## 4 DISCUSSION

Placental trophoblasts serve several critical roles in the development of the fetus. During mammalian development, the placental cytotrophoblasts lining the microvilli fuse to form syncytiotrophoblasts, which regulate oxygen and nutrient flow from maternal blood to the fetus while carbon dioxide and metabolic wastes are eliminated from the fetus to maternal blood. Thus, syncytia formation is a highly specialized and essential process in mammalian placental development.

Within the mature syncytiotrophoblast, TFEB is one of the most highly expressed proteins. Using the human BeWo cytotrophoblast cells, which fuse to form multinucleate syncytiotrophoblasts upon exposure to forskolin, we have characterized the subcellular localization, effects of nutrient starvation, effects of mTOR regulation, and phosphorylation state on cellular TFEB. Further, we have determined the degradation rate of endogenous TFEB and the mechanism thereof in both cytotrophoblasts and syncytiotrophoblasts.

In BeWo cytotrophoblasts and syncytiotrophoblasts, the subcellular distribution of TFEB is strikingly different. TFEB is localized largely to the cytoplasm in cytotrophoblasts and largely to the nucleus in syncytiotrophoblasts (Figures 1 and 2). This expression pattern is seen in both natural syncytia (in the absence of forskolin) and in forskolin‐induced syncytia (Figure 1). Cesana et al. (2024) have also demonstrated TFEB translocation to the nucleus during syncytiotrophoblast development.

Nonetheless, in cytotrophoblasts similar to other cell types including HeLa and HEK293T (Napolitano et al., 2018; Tan et al., 2022), TFEB translocates to the nucleus under conditions of amino acid starvation and returns to the cytoplasm upon refeeding (Figure 4). Phosphorylation of serine residues at positions 122, 142, and 211 has been shown to be responsible. As expected, on Western blots of cytotrophoblasts and syncytotrophoblasts during amino acid starvation, the lower apparent molecular weight forms of TFEB are nuclear (Figure 4). These forms are not recognized with anti‐phospho‐TFEB antibodies, whereas the higher apparent molecular weight forms in the post‐nuclear supernatant (i.e., cytoplasm) are recognized with anti‐phospho (122, 211) TFEB antibodies (Figure 3).

mTOR‐mediated phosphorylation of TFEB occurs at serines 122, 142, and 211 (Napolitano & Ballabio, 2016; Puertollano et al., 2018; Settembre et al., 2011; Vega‐Rubin‐de‐Celis et al., 2017) with 122 and 211 generally dominant (Livneh et al., 2023). As noted above, phospho‐TFEB is cytoplasmic. Mutation of serine 211 to alanine results in constitutively nuclear TFEB similar to that seen in cells treated with the mTOR inhibitor, Torin (Martina et al., 2012; Roczniak‐Ferguson et al., 2012; Settembre et al., 2012). In addition, serine 138 phosphorylation, which is mTOR‐independent, also plays a role in nuclear TFEB translocation. Recently, Napolitano et al. (2018) demonstrated dynamic and continuous shuttling of TFEB into and out of the nucleus. Nuclear export is mediated via CRM1/Exportin1, the rate‐limiting step, and modulated via nutrient availability and phosphorylation at serines 142 and 138. Consistent with this, both nutrient (amino acid) starvation and mTOR inhibition with Torin resulted in nuclear accumulation of TFEB in cytotrophoblasts and in syncytiotrophoblasts (Figure 5). Taken together, these observations demonstrate the subcellular localization and behavior of TFEB in trophoblasts. Indeed, recently, Cesana et al. (2024) showed that TFEB knockout mice display defective syncytiotrophoblast formation, and consistent with that, in vitro, forskolin treatment induced binding of TFEB to promoters of Syncytin‐1 and Syncytin‐2.

While the activity of TFEB is regulated in part via phosphorylation and nuclear‐cytoplasmic shuttling, TFEB cellular abundance is another determinant of TFEB function. Many transcription factors are short‐lived proteins. Short‐lived proteins generally have half‐lives of hours or less. Using four human cell lines and cycloheximide chase assays combined with quantitative proteomics, Li et al. (2021) identified 1017 short‐lived (*t*_1/2_ less than/equal to 8 h) proteins among the 11,747 quantified proteins. The median half‐lives of the short‐lived proteins were 3–4 h with ~100 proteins having half‐lives less than or equal to 2 h. Sha et al. (2017) have shown that TFEB is degraded via the ubiquitin proteasome system in HeLa (t_1/2_ ~ 6 h), HEK, and MEF cells and that STUB1 serves as an E3 ubiquitin ligase preferentially targeting phosphorylated (inactive) TFEB for degradation. We examined endogenous TFEB degradation in cytotrophoblasts and syncytiotrophoblasts in cycloheximide chase experiments (Figure 6). Herein, TFEB was degraded with half‐lives of 2–3 h in both cytotrophoblasts and syncytiotrophoblasts and was inhibited by the presence of MG132, a potent proteasomal inhibitor. Phospho‐TFEB was degraded with half‐lives of 1.8–2.2 h in both cytotrophoblasts and syncytiotrophoblasts and was stabilized by MG132. The degradation rate of phospho‐TFEB (*t*_1/2_ ~ 2.2 h) was slightly more rapid than that of TFEB (*t*_1/2_ ~ 3.0 h) in the cytotrophoblasts. The degradation rate of phospho‐TFEB (*t*_1/2_ ~ 1.8 h) was also slightly more rapid than that of TFEB (*t*_1/2_ ~ 2.6 h) in syncytiotrophoblasts. Of note is that the degradation rate of TFEB was very similar whether TFEB was largely in the cytoplasm (in cytotrophoblasts) or within the nucleus (in syncytiotrophoblasts). This is not entirely surprising in that the ubiquitin proteasome degradation system exists and functions within both the cell cytoplasm and nucleus (Enam et al., 2018; Floyd et al., 2001; Franić et al., 2021; Lingbeck et al., 2003; Livneh et al., 2023). Yet, as discussed above, TFEB within the nucleus is unphosphorylated, whereas TFEB within the cytoplasm is largely phosphorylated. Furthermore, the E3 ubiquitin ligase STUB1 (also known as CHIP) is widely expressed. In placenta, STUB1 is present in cytotrophoblasts and syncytiotrophoblasts, in both the cytoplasm and nucleus (see Human Protein Atlas, (Fagerberg et al., 2014)). Thus, it remains unclear as to whether STUB1 is the only E3 ubiquitin ligase responsible for TFEB degradation in trophoblasts and whether TFEB is exported from the nucleus prior to degradation within the cytoplasm. Recently, Livneh et al. (2023) have shown that the proteasome is exported from the nucleus via the CRM1/Exportin1 pathway. One potentially significant difference in our results and those of Sha et al. (2017) has to do with the TFEB substrate examined in the degradation experiments. Sha et al. (2017) examined Flag‐TFEB and GFP‐TFEB, while we examined native endogenous TFEB. It should be noted that many ubiquitin proteasome system substrates are recognized via E3 ubiquitin ligases at their free N‐termini (e.g., MyoD, Id1, p16INK4a, HPV58‐E7; (Breitschopf et al., 1998; Ciechanover & Ben‐Saadon, 2004; Trausch‐Azar et al., 2004)). Future studies of TFEB degradation will be necessary to define the TFEB motifs essential for ubiquitin proteasome recognition and degradation. Furthermore, identifying the role of trophoblast nuclear import and export of both TFEB and the proteasome will be necessary to clarify these issues. Limitations of the present studies herein include the observational studies in Figures 4 and 5 (*n* = 2) and the use of supra‐physiological D‐glucose concentrations in the culture of both HeLa and BeWo cells. In addition, phospho‐TFEB was determined only with specific antibodies to TFEB with phospho‐Ser122 and with phospho‐Ser211. Future studies will also examine TFEB biology in freshly isolated and cultured primary human cytotrophoblasts.

In addition to those discussed above, additional mechanisms may be involved in the subcellular physiology of TFEB including protein liquid–liquid phase separation (LLPS) (Banani et al., 2017; Chen et al., 2020; Kaganovich, 2017). (Wang et al., 2022) demonstrated that nuclear TFEB forms distinct puncta and that inositol polyphosphate multikinase inhibits LLPS of TFEB. Furthermore, while liquid‐like condensates of TFEB exhibit low fusion propensity in living cells, a variety of chemical effectors modulate these properties and may mediate TFEB functions independent of alterations in cytoplasmic‐nuclear translocation. Importantly, Fu et al. (2021) have described many of the dynamic properties of nuclear condensates, a subset of which serve as central foci for the entire ubiquitin proteasome enzymatic machinery and are essential for degradation of selective nuclear proteins. In addition, they showed that nuclear condensates are involved in the degradation of misfolded proteins via the recruitment of STUB1 (Fu et al., 2021). Most recently, Livneh et al. (2023) have shown that nucleo‐cytoplasmic shuttling of the proteasome is under exquisite regulation whereby aromatic amino acids (YWF) promote proteasome translocation from its large nuclear pool to the cytoplasm. Thus, whether LLPS physiology together with YWF‐mediated mTOR signaling modulates cytoplasmic and/or nuclear degradation of TFEB remains to be elucidated.

## AUTHOR CONTRIBUTIONS

Schwartz A.L., Mathew A., Trausch‐Azar J.S., Mahjoub M.R. designed experiments; all authors performed experiments and analyzed data; Mathew A. and Schwartz A.L. were primary authors of the manuscript; all authors finalized the manuscript.

## FUNDING INFORMATION

None.

## CONFLICTS OF INTEREST

The authors declare no conflicts of interest.

## ETHICS STATEMENT

None.

## Supporting information


**Figure S1.** Expression of hCGβ in BeWo Cell Lysates ± Forskolin. BeWo cells were grown in culture for 72 h in the absence or presence of forskolin as described in Methods. Thereafter, three independent cultures had cell lysates prepared and were analyzed via Western blot with anti‐hCGβ. HeLa cell lysates were used as a negative control.
**Figure S2.** TFEB Localization in Cell Fractions of HeLa Cells. HeLa cells were harvested following which whole cell extract (WCE), post‐nuclear supernatant extract (PNS, cytoplasm) and nuclear extract (NE) were prepared and analyzed via Western blot with anti‐TFEB antibody. Controls of anti‐lamin B1 and anti‐alpha tubulin are shown. The 75 kDa marker is indicated by closed triangle. The open triangle denotes lower molecular weight (unphosphorylated) TFEB.
**Figure S3.** TFEB Localization Following Torin Treatment in HeLa Cells. Cells were incubated without or with 250 nM Torin, as indicated and thereafter were harvested, fractionated, run on Western blots, and probed with anti‐TFEB as in Figure S2. Controls and markers are as in Figure 2.
**Figure S4.** Phospho‐TFEB and Total TFEB in BeWo Cytotrophoblasts and Syncytiotrophoblasts under Fed and Starved Conditions. BeWo cells were incubated for 72 h ± forskolin after which they were incubated with complete media for 2 h (fed) and for an additional 1 h with EBSS (starved) as indicated. Thereafter cells were harvested for Western Blot analysis. Western blots were initially probed with anti‐TFEB plus anti‐actin and thereafter stripped and reprobed with anti‐phospho‐TFEB plus anti‐actin as in Figure 3. The 75 kDa marker is indicated by the closed triangle. The open triangle denotes lower molecular weight (unphosphorylated) TFEB. The actin band is also indicated. For each of the four experimental conditions the mean of total TFEB density units/actin units was set to 100% and the percent phospho‐TFEB of total TFEB was calculated. Data in the bar graphs are indicated as mean ± SEM of 3 experiments with individual dots for each sample and the mean as a bar. Statistical comparisons of total TFEB and phospho‐TFEB are displayed above the bar graphs for each of the four conditions.
**Figure S5.** Degradation of Endogenous TFEB in HeLa Cells. Cells were incubated with 100 μg/mL cycloheximide (CHX) in the absence or presence of 20 μM MG132. At 0, 2, 4 and 6 h (CHX) and 0, 4 and 6 h (CHX + MG132) cells were harvested and analyzed via Western blot with anti‐TFEB antibody. The TFEB was quantified as described in Methods and displayed in the Figure, CHX alone (●), CHX + MG132 (■). Half‐lives were calculated as indicated (mean ± SEM of 3 independent experiments). Upper panels are representative Western blots with anti‐TFEB and anti‐actin, as control.

## Data Availability

No datasets were generated or analyzed during the current study. Reasonable requests for included data will be provided by the senior author.
