# Supplementary material for: Regulation of TFEB in human placental Cytotrophoblasts and Syncytiotrophoblasts
Source: Physiol Rep. 2025 May 26;13(10):e70383. doi: 10.14814/phy2.70383 (PMC12104664; doi:10.14814/phy2.70383)
Supplement: Supplementary file 1 — Figure S1. Expression of hCGβ in BeWo Cell Lysates ± Forskolin. BeWo cells were grown in culture for 72 h in the absence or presence of forskolin as described in Methods. Thereafter, three independent cultures had cell lysates prepared and were analyzed via Western blot with anti‐hCGβ. HeLa cell lysates were used as a negative control. Figure S2. TFEB Localization in Cell Fractions of HeLa Cells. HeLa cells were harvested following which whole cell extract (WCE), post‐nuclear supernatant extract (PNS, cytoplasm) and nuclear extract (NE) were prepared and analyzed via Western blot with anti‐TFEB antibody. Controls of anti‐lamin B1 and anti‐alpha tubulin are shown. The 75 kDa marker is indicated by closed triangle. The open triangle denotes lower molecular weight (unphosphorylated) TFEB. Figure S3. TFEB Localization Following Torin Treatment in HeLa Cells. Cells were incubated without or with 250 nM Torin, as indicated and thereafter were harvested, fractionated, run on Western blots, and probed with anti‐TFEB as in Figure S2. Controls and markers are as in Figure 2. Figure S4. Phospho‐TFEB and Total TFEB in BeWo Cytotrophoblasts and Syncytiotrophoblasts under Fed and Starved Conditions. BeWo cells were incubated for 72 h ± forskolin after which they were incubated with complete media for 2 h (fed) and for an additional 1 h with EBSS (starved) as indicated. Thereafter cells were harvested for Western Blot analysis. Western blots were initially probed with anti‐TFEB plus anti‐actin and thereafter stripped and reprobed with anti‐phospho‐TFEB plus anti‐actin as in Figure 3. The 75 kDa marker is indicated by the closed triangle. The open triangle denotes lower molecular weight (unphosphorylated) TFEB. The actin band is also indicated. For each of the four experimental conditions the mean of total TFEB density units/actin units was set to 100% and the percent phospho‐TFEB of total TFEB was calculated. Data in the bar graphs are indicated as mean ± SEM of 3 experiments with [file PHY2-13-e70383-s001.pdf]

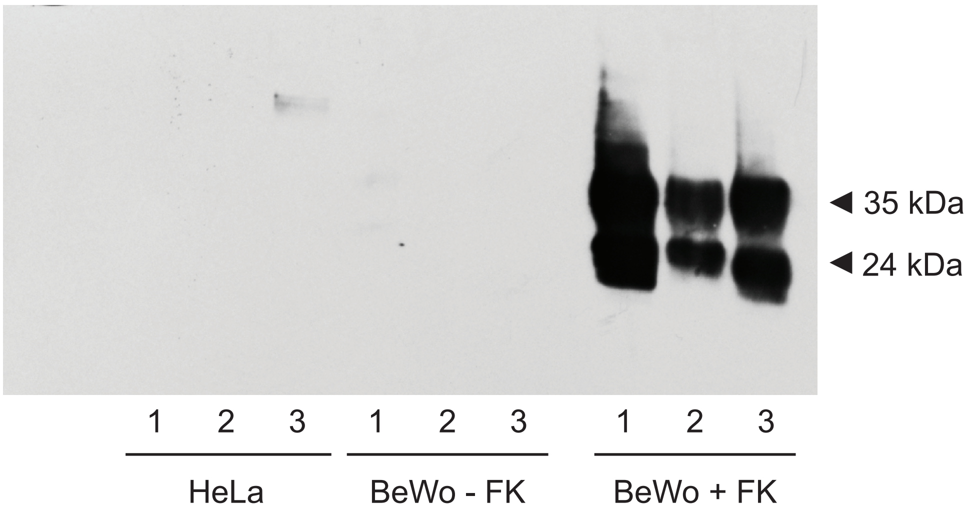

Supplementary Figure 1

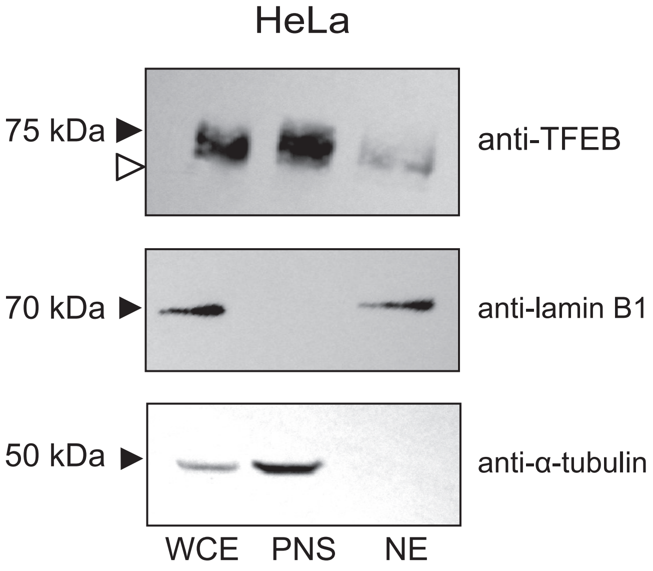

Supplementary Figure 2

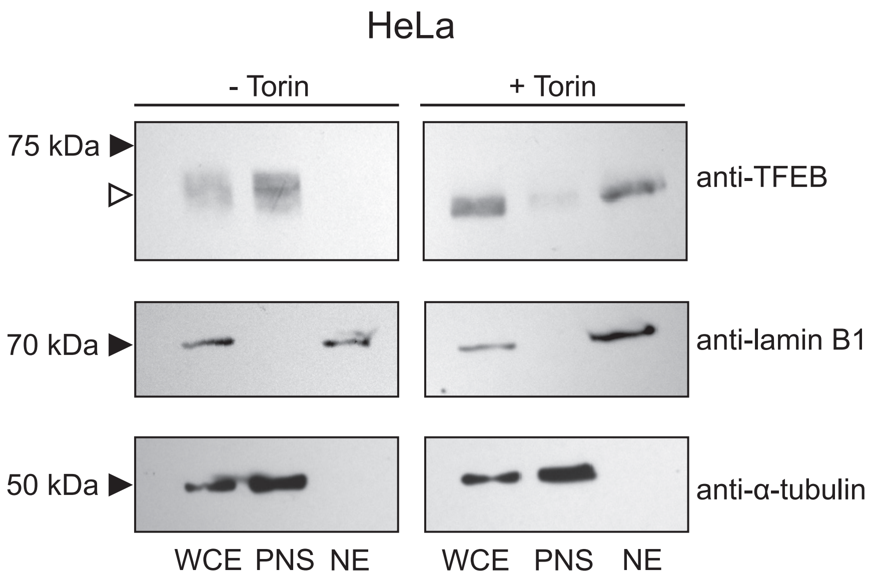

Supplementary Figure 3

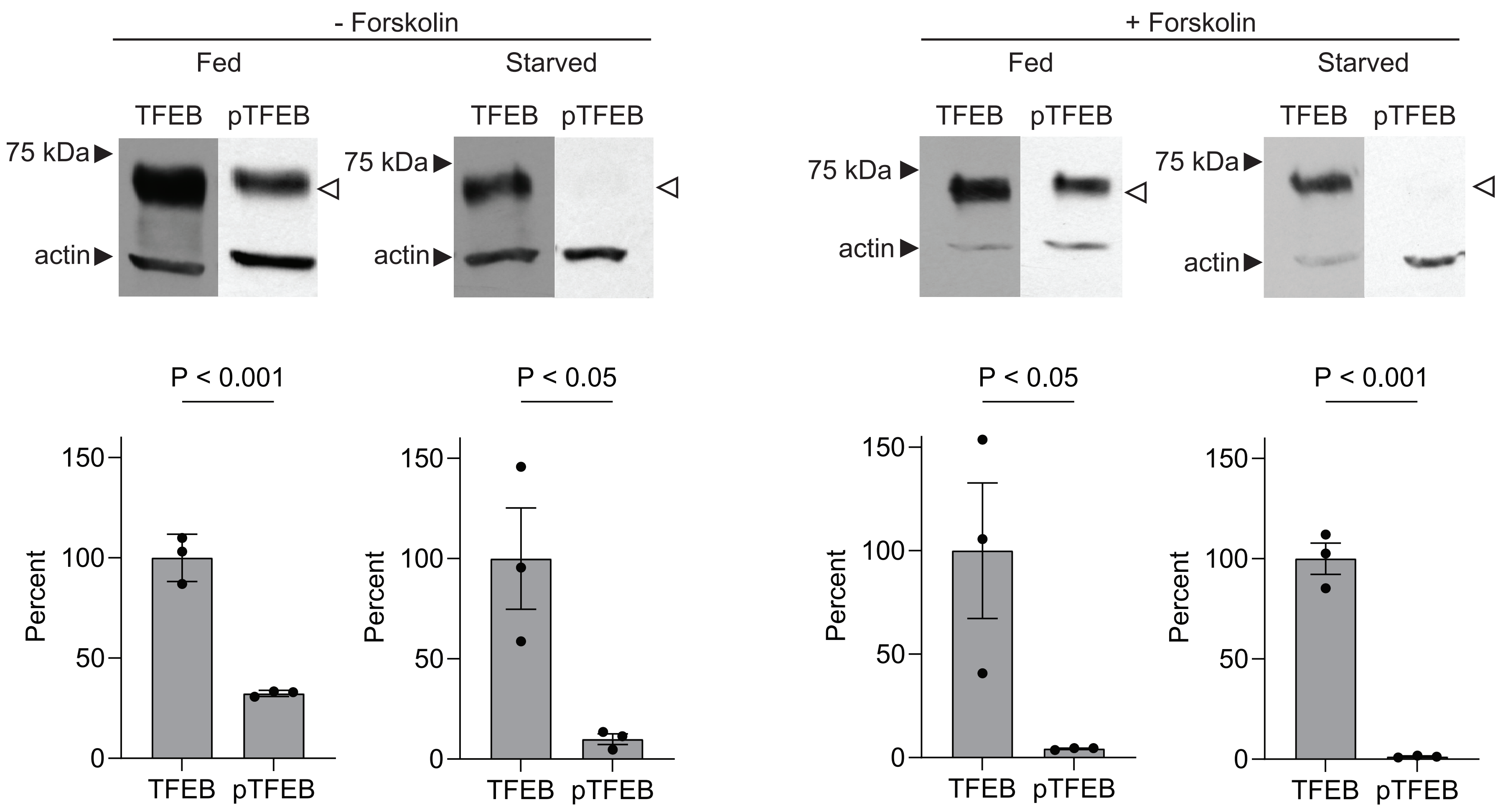

Supplementary Figure 4

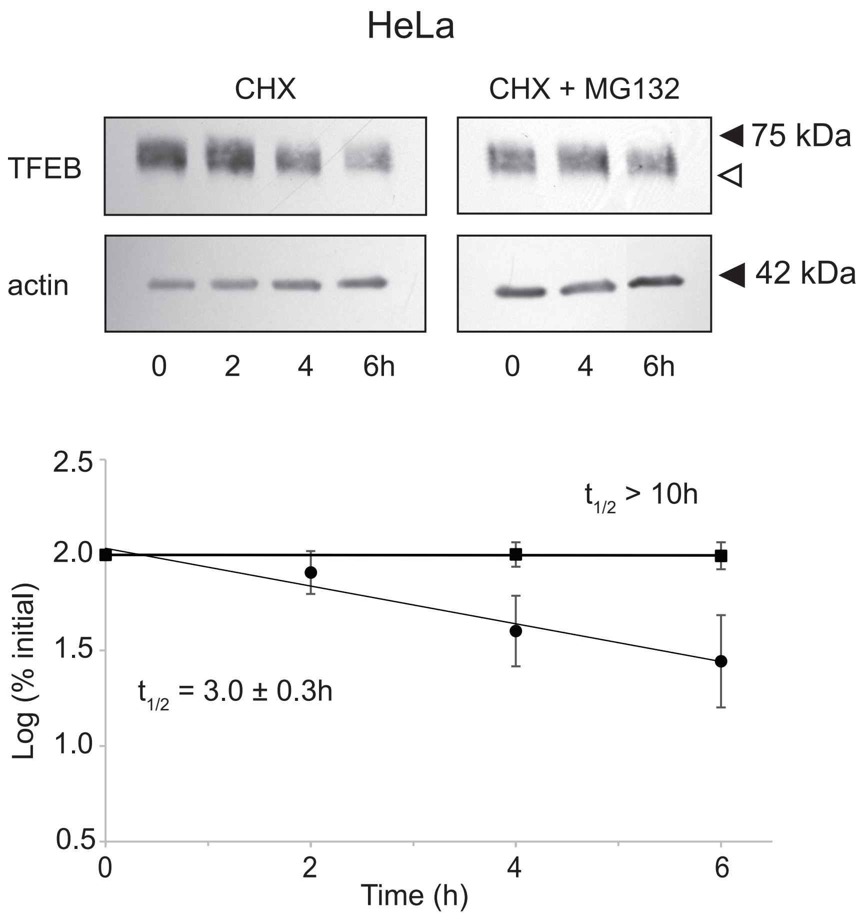

Supplementary Figure 5
